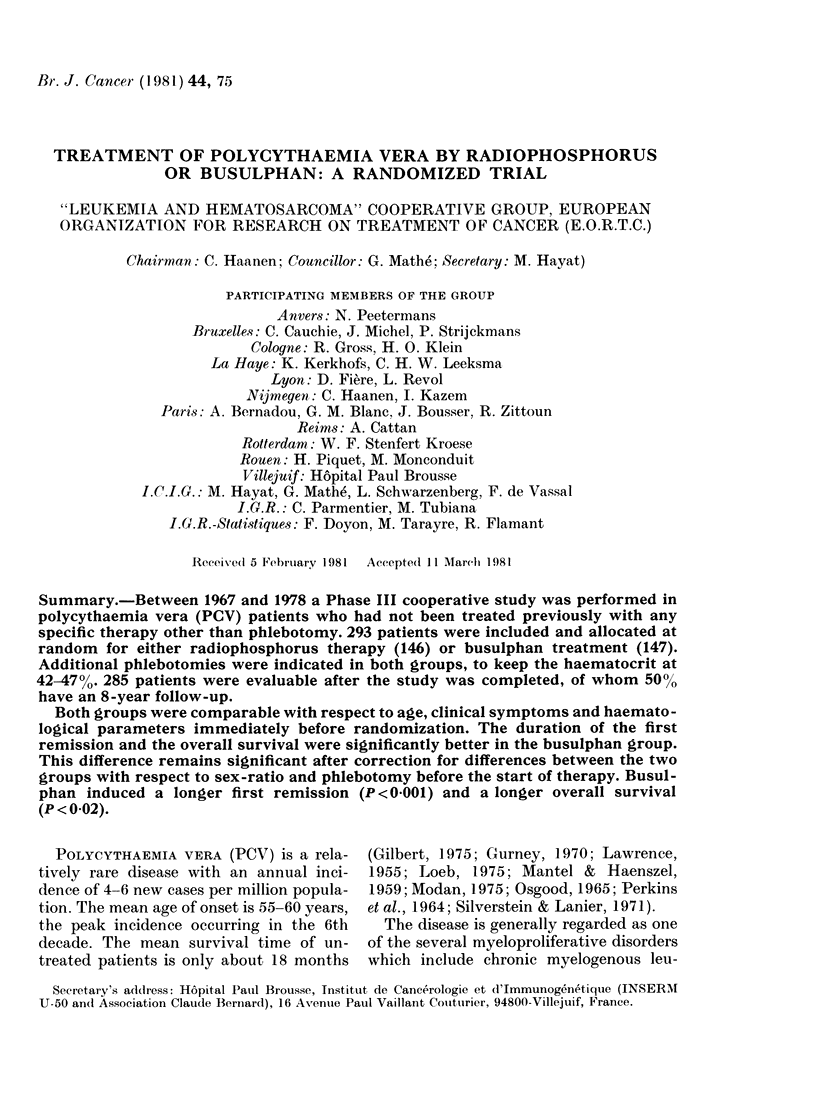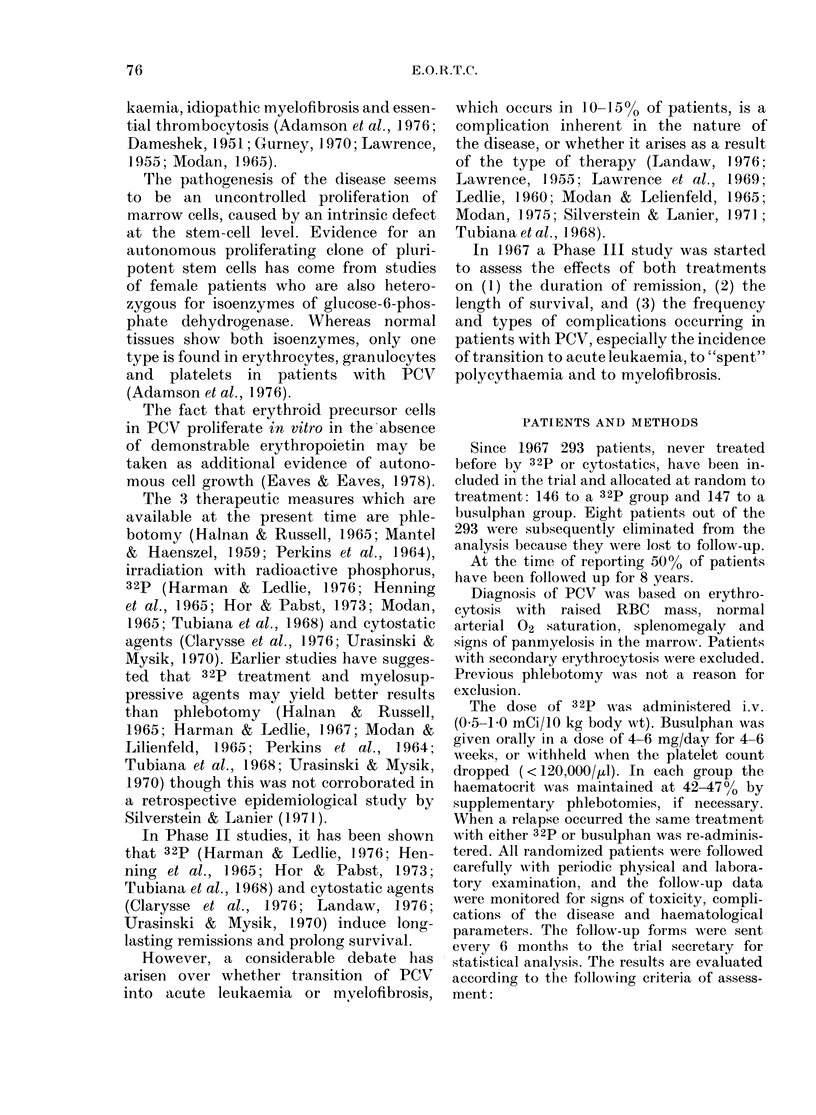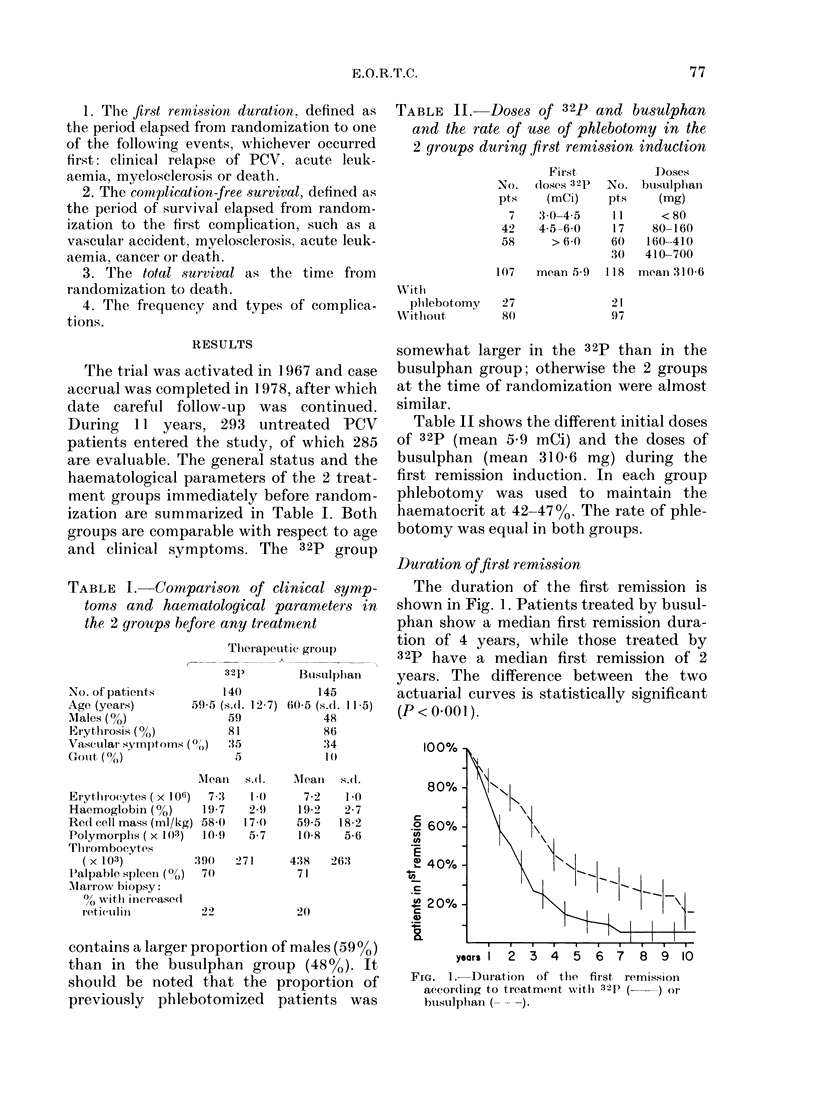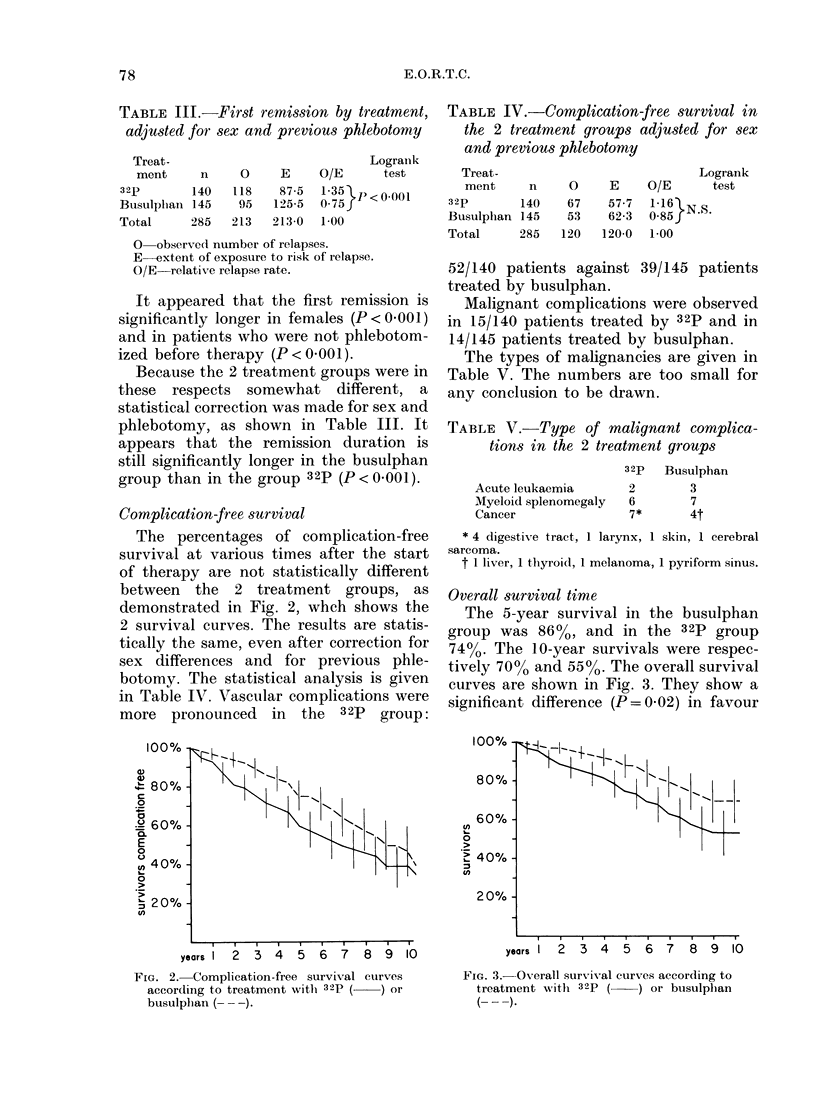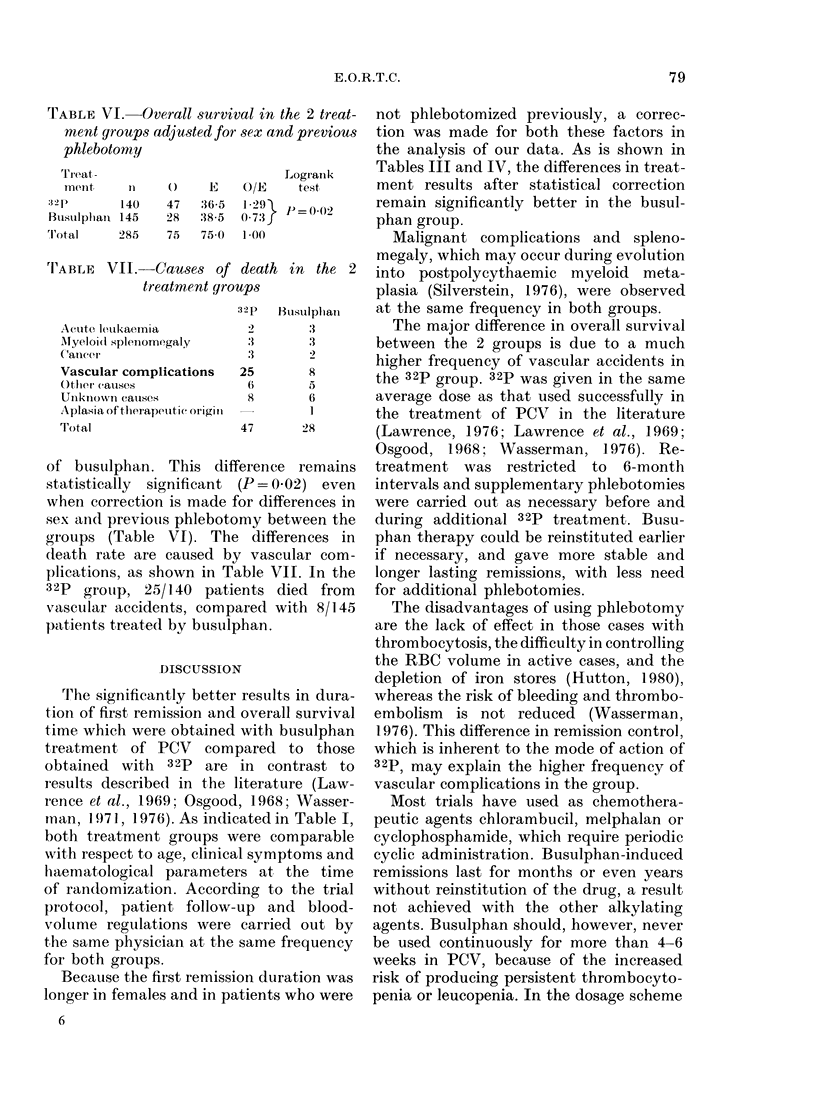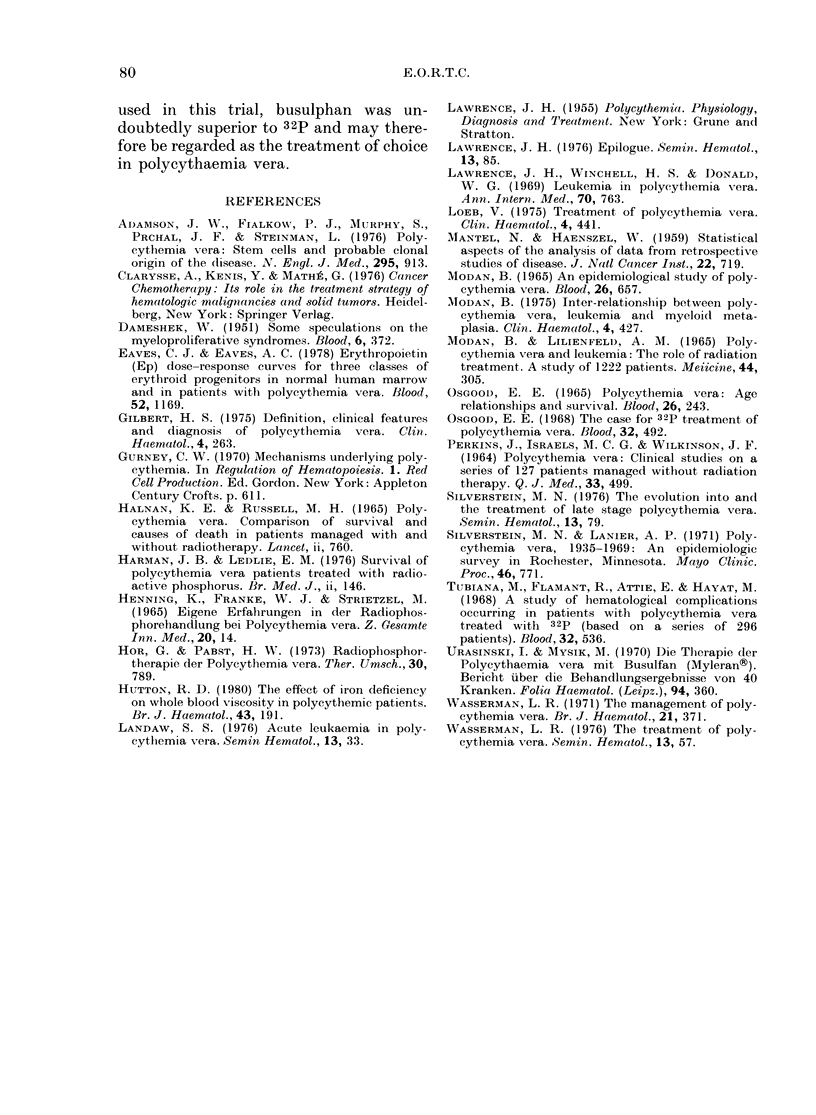# Treatment of polycythaemia vera by radiophosphorus or busulphan: a randomized trial. "Leukemia and Hematosarcoma" Cooperative Group, European Organization for Research on Treatment of Cancer (E.O.R.T.C.).

**DOI:** 10.1038/bjc.1981.150

**Published:** 1981-07

**Authors:** 

## Abstract

Between 1967 and 1978 a Phase III cooperative study was performed in polycythaemia vera (PCV) patients who had not been treated previously with any specific therapy other than phlebotomy. 293 patients were included and allocated at random for either radiophosphorus therapy (146) or busulphan treatment (147). Additional phlebotomies were indicated in both groups, to keep the haematocrit at 42-47%. 285 patients were evaluable after the study was completed, of whom 50% have an 8-year follow-up. Both groups were comparable with respect to age, clinical symptoms and haematological parameters immediately before randomization. The duration of the first remission and the overall survival were significantly better in the busulphan group. This difference remains significant after correction for differences between the two groups with respect to sex-ratio and phlebotomy before and the start of therapy. Busulphan induced a longer first remission (P less than 0.001) and a longer overall survival (P less than 0.02).


*Br. J. Cancer* (1981) **44**, 75

### TREATMENT OF POLYCYTHAEMIA VERA BY RADIOPHOSPHORUS OR BUSULPHAN: A RANDOMIZED TRIAL

"LEUKEMIA AND HEMATOSARCOMA" COOPERATIVE GROUP, EUROPEAN ORGANIZATION FOR RESEARCH ON TREATMENT OF CANCER (E.O.R.T.C.)

*Chairman:* C. Haanen; *Councillor:* G. Mathé; *Secretary:* M. Hayat)

PARTICIPATING MEMBERS OF THE GROUP

*Anvers:* N. Peetermans
*Bruxelles:* C. Cauchie, J. Michel, P. Strijckmans
*Cologne:* R. Gross, H. O. Klein
*La Haye:* K. Kerkhofs, C. H. W. Leeksma
*Lyon:* D. Fière, L. Revol
*Nijmegen:* C. Haanen, I. Kazem
*Paris:* A. Bernadou, G. M. Blanc, J. Bousser, R. Zittoun
*Reims:* A. Cattan
*Rotterdam:* W. F. Stenfert Kroese
*Rouen:* H. Piquet, M. Monconduit
*Villejuif:* Hôpital Paul Brousse
*I.C.I.G.:* M. Hayat, G. Mathé, L. Schwarzenberg, F. de Vassal
*I.G.R.:* C. Parmentier, M. Tubiana
*I.G.R.-Statistiques:* F. Doyon, M. Tarayre, R. Flamant

Received 5 February 1981 Accepted 11 March 1981

**Summary.—Between 1967 and 1978 a Phase III cooperative study was performed in polycythaemia vera (PCV) patients who had not been treated previously with any specific therapy other than phlebotomy. 293 patients were included and allocated at random for either radiophosphorus therapy (146) or busulphan treatment (147). Additional phlebotomies were indicated in both groups, to keep the haematocrit at 42–47%. 285 patients were evaluable after the study was completed, of whom 50% have an 8-year follow-up.**

**Both groups were comparable with respect to age, clinical symptoms and haematological parameters immediately before randomization. The duration of the first remission and the overall survival were significantly better in the busulphan group. This difference remains significant after correction for differences between the two groups with respect to sex-ratio and phlebotomy before the start of therapy. Busulphan induced a longer first remission ($P<0.001$) and a longer overall survival ($P<0.02$).**

Polycythaemia vera (PCV) is a relatively rare disease with an annual incidence of 4–6 new cases per million population. The mean age of onset is 55–60 years, the peak incidence occurring in the 6th decade. The mean survival time of untreated patients is only about 18 months (Gilbert, 1975; Gurney, 1970; Lawrence, 1955; Loeb, 1975; Mantel & Haenszel, 1959; Modan, 1975; Osgood, 1965; Perkins *et al.*, 1964; Silverstein & Lanier, 1971).

The disease is generally regarded as one of the several myeloproliferative disorders which include chronic myelogenous leu-

Secretary's address: Hôpital Paul Brousse, Institut de Cancérologie et d'Immunogénétique (INSERM U-50 and Association Claude Bernard), 16 Avenue Paul Vaillant Couturier, 94800-Villejuif, France.

kaemia, idiopathic myelofibrosis and essential thrombocytosis (Adamson *et al.*, 1976; Dameshek, 1951; Gurney, 1970; Lawrence, 1955; Modan, 1965).

The pathogenesis of the disease seems to be an uncontrolled proliferation of marrow cells, caused by an intrinsic defect at the stem-cell level. Evidence for an autonomous proliferating clone of pluripotent stem cells has come from studies of female patients who are also heterozygous for isoenzymes of glucose-6-phosphate dehydrogenase. Whereas normal tissues show both isoenzymes, only one type is found in erythrocytes, granulocytes and platelets in patients with PCV (Adamson *et al.*, 1976).

The fact that erythroid precursor cells in PCV proliferate *in vitro* in the absence of demonstrable erythropoietin may be taken as additional evidence of autonomous cell growth (Eaves & Eaves, 1978).

The 3 therapeutic measures which are available at the present time are phlebotomy (Halnan & Russell, 1965; Mantel & Haenszel, 1959; Perkins *et al.*, 1964), irradiation with radioactive phosphorus, $^{32}P$ (Harman & Ledlie, 1976; Henning *et al.*, 1965; Hor & Pabst, 1973; Modan, 1965; Tubiana *et al.*, 1968) and cytostatic agents (Clarysse *et al.*, 1976; Urasinski & Mysik, 1970). Earlier studies have suggested that $^{32}P$ treatment and myelosuppressive agents may yield better results than phlebotomy (Halnan & Russell, 1965; Harman & Ledlie, 1967; Modan & Lilienfeld, 1965; Perkins *et al.*, 1964; Tubiana *et al.*, 1968; Urasinski & Mysik, 1970) though this was not corroborated in a retrospective epidemiological study by Silverstein & Lanier (1971).

In Phase II studies, it has been shown that $^{32}P$ (Harman & Ledlie, 1976; Henning *et al.*, 1965; Hor & Pabst, 1973; Tubiana *et al.*, 1968) and cytostatic agents (Clarysse *et al.*, 1976; Landaw, 1976; Urasinski & Mysik, 1970) induce long-lasting remissions and prolong survival.

However, a considerable debate has arisen over whether transition of PCV into acute leukaemia or myelofibrosis, which occurs in 10–15% of patients, is a complication inherent in the nature of the disease, or whether it arises as a result of the type of therapy (Landaw, 1976; Lawrence, 1955; Lawrence *et al.*, 1969; Ledlie, 1960; Modan & Lelienfeld, 1965; Modan, 1975; Silverstein & Lanier, 1971; Tubiana *et al.*, 1968).

In 1967 a Phase III study was started to assess the effects of both treatments on (1) the duration of remission, (2) the length of survival, and (3) the frequency and types of complications occurring in patients with PCV, especially the incidence of transition to acute leukaemia, to "spent" polycythaemia and to myelofibrosis.

### PATIENTS AND METHODS

Since 1967 293 patients, never treated before by $^{32}P$ or cytostatics, have been included in the trial and allocated at random to treatment: 146 to a $^{32}P$ group and 147 to a busulphan group. Eight patients out of the 293 were subsequently eliminated from the analysis because they were lost to follow-up.

At the time of reporting 50% of patients have been followed up for 8 years.

Diagnosis of PCV was based on erythrocytosis with raised RBC mass, normal arterial $O_2$ saturation, splenomegaly and signs of panmyelosis in the marrow. Patients with secondary erythrocytosis were excluded. Previous phlebotomy was not a reason for exclusion.

The dose of $^{32}P$ was administered i.v. (0·5–1·0 mCi/10 kg body wt). Busulphan was given orally in a dose of 4–6 mg/day for 4–6 weeks, or withheld when the platelet count dropped ($<120{,}000/\mu l$). In each group the haematocrit was maintained at 42–47% by supplementary phlebotomies, if necessary. When a relapse occurred the same treatment with either $^{32}P$ or busulphan was re-administered. All randomized patients were followed carefully with periodic physical and laboratory examination, and the follow-up data were monitored for signs of toxicity, complications of the disease and haematological parameters. The follow-up forms were sent every 6 months to the trial secretary for statistical analysis. The results are evaluated according to the following criteria of assessment:

1. The *first remission duration*, defined as the period elapsed from randomization to one of the following events, whichever occurred first: clinical relapse of PCV, acute leukaemia, myelosclerosis or death.

2. The *complication-free survival*, defined as the period of survival elapsed from randomization to the first complication, such as a vascular accident, myelosclerosis, acute leukaemia, cancer or death.

3. The *total survival* as the time from randomization to death.

4. The frequency and types of complications.

### RESULTS

The trial was activated in 1967 and case accrual was completed in 1978, after which date careful follow-up was continued. During 11 years, 293 untreated PCV patients entered the study, of which 285 are evaluable. The general status and the haematological parameters of the 2 treatment groups immediately before randomization are summarized in Table I. Both groups are comparable with respect to age and clinical symptoms. The $^{32}P$ group contains a larger proportion of males (59%) than in the busulphan group (48%). It should be noted that the proportion of previously phlebotomized patients was somewhat larger in the $^{32}P$ than in the busulphan group; otherwise the 2 groups at the time of randomization were almost similar.

TABLE I.—*Comparison of clinical symptoms and haematological parameters in the 2 groups before any treatment*

| | Therapeutic group: $^{32}P$ | | Therapeutic group: Busulphan | |
|---|---|---|---|---|
| No. of patients | 140 | | 145 | |
| Age (years) | 59·5 (s.d. 12·7) | | 60·5 (s.d. 11·5) | |
| Males (%) | 59 | | 48 | |
| Erythrosis (%) | 81 | | 86 | |
| Vascular symptoms (%) | 35 | | 34 | |
| Gout (%) | 5 | | 10 | |
| | Mean | s.d. | Mean | s.d. |
| Erythrocytes ($\times 10^6$) | 7·3 | 1·0 | 7·2 | 1·0 |
| Haemoglobin (%) | 19·7 | 2·9 | 19·2 | 2·7 |
| Red cell mass (ml/kg) | 58·0 | 17·0 | 59·5 | 18·2 |
| Polymorphs ($\times 10^3$) | 10·9 | 5·7 | 10·8 | 5·6 |
| Thrombocytes ($\times 10^3$) | 390 | 271 | 438 | 263 |
| Palpable spleen (%) | 70 | | 71 | |
| Marrow biopsy: % with increased reticulin | 22 | | 20 | |

TABLE II.—*Doses of $^{32}P$ and busulphan and the rate of use of phlebotomy in the 2 groups during first remission induction*

| | No. pts | First doses $^{32}P$ (mCi) | No. pts | Doses busulphan (mg) |
|---|---|---|---|---|
| | 7 | 3·0–4·5 | 11 | < 80 |
| | 42 | 4·5–6·0 | 17 | 80–160 |
| | 58 | > 6·0 | 60 | 160–410 |
| | | | 30 | 410–700 |
| | 107 | mean 5·9 | 118 | mean 310·6 |
| With phlebotomy | 27 | | 21 | |
| Without | 80 | | 97 | |

Table II shows the different initial doses of $^{32}P$ (mean 5·9 mCi) and the doses of busulphan (mean 310·6 mg) during the first remission induction. In each group phlebotomy was used to maintain the haematocrit at 42–47%. The rate of phlebotomy was equal in both groups.

### *Duration of first remission*

The duration of the first remission is shown in Fig. 1. Patients treated by busulphan show a median first remission duration of 4 years, while those treated by $^{32}P$ have a median first remission of 2 years. The difference between the two actuarial curves is statistically significant ($P < 0{\cdot}001$).

FIG. 1.—Duration of the first remission according to treatment with $^{32}P$ (——) or busulphan (– – –).

TABLE III.—*First remission by treatment, adjusted for sex and previous phlebotomy*

| Treatment | n | O | E | O/E | Logrank test |
|---|---|---|---|---|---|
| $^{32}P$ | 140 | 118 | 87·5 | 1·35 | $P < 0{\cdot}001$ |
| Busulphan | 145 | 95 | 125·5 | 0·75 | |
| Total | 285 | 213 | 213·0 | 1·00 | |

O—observed number of relapses.
E—extent of exposure to risk of relapse.
O/E—relative relapse rate.

It appeared that the first remission is significantly longer in females ($P < 0{\cdot}001$) and in patients who were not phlebotomized before therapy ($P < 0{\cdot}001$).

Because the 2 treatment groups were in these respects somewhat different, a statistical correction was made for sex and phlebotomy, as shown in Table III. It appears that the remission duration is still significantly longer in the busulphan group than in the group $^{32}P$ ($P < 0{\cdot}001$).

*Complication-free survival*

The percentages of complication-free survival at various times after the start of therapy are not statistically different between the 2 treatment groups, as demonstrated in Fig. 2, whch shows the 2 survival curves. The results are statistically the same, even after correction for sex differences and for previous phlebotomy. The statistical analysis is given in Table IV. Vascular complications were more pronounced in the $^{32}P$ group: 52/140 patients against 39/145 patients treated by busulphan.

TABLE IV.—*Complication-free survival in the 2 treatment groups adjusted for sex and previous phlebotomy*

| Treatment | n | O | E | O/E | Logrank test |
|---|---|---|---|---|---|
| $^{32}P$ | 140 | 67 | 57·7 | 1·16 | N.S. |
| Busulphan | 145 | 53 | 62·3 | 0·85 | |
| Total | 285 | 120 | 120·0 | 1·00 | |

Malignant complications were observed in 15/140 patients treated by $^{32}P$ and in 14/145 patients treated by busulphan.

The types of malignancies are given in Table V. The numbers are too small for any conclusion to be drawn.

TABLE V.—*Type of malignant complications in the 2 treatment groups*

| | $^{32}P$ | Busulphan |
|---|---|---|
| Acute leukaemia | 2 | 3 |
| Myeloid splenomegaly | 6 | 7 |
| Cancer | 7* | 4† |

* 4 digestive tract, 1 larynx, 1 skin, 1 cerebral sarcoma.
† 1 liver, 1 thyroid, 1 melanoma, 1 pyriform sinus.

*Overall survival time*

The 5-year survival in the busulphan group was 86%, and in the $^{32}P$ group 74%. The 10-year survivals were respectively 70% and 55%. The overall survival curves are shown in Fig. 3. They show a significant difference ($P = 0{\cdot}02$) in favour

FIG. 2.—Complication-free survival curves according to treatment with $^{32}P$ (——) or busulphan (– – –).

FIG. 3.—Overall survival curves according to treatment with $^{32}P$ (——) or busulphan (– – –).

TABLE VI.—*Overall survival in the 2 treatment groups adjusted for sex and previous phlebotomy*

| Treatment | n | O | E | O/E | Logrank test |
|---|---|---|---|---|---|
| $^{32}P$ | 140 | 47 | 36·5 | 1·29 | $P=0·02$ |
| Busulphan | 145 | 28 | 38·5 | 0·73 | |
| Total | 285 | 75 | 75·0 | 1·00 | |

TABLE VII.—*Causes of death in the 2 treatment groups*

| | $^{32}P$ | Busulphan |
|---|---|---|
| Acute leukaemia | 2 | 3 |
| Myeloid splenomegaly | 3 | 3 |
| Cancer | 3 | 2 |
| **Vascular complications** | **25** | 8 |
| Other causes | 6 | 5 |
| Unknown causes | 8 | 6 |
| Aplasia of therapeutic origin | — | 1 |
| Total | 47 | 28 |

of busulphan. This difference remains statistically significant ($P=0·02$) even when correction is made for differences in sex and previous phlebotomy between the groups (Table VI). The differences in death rate are caused by vascular complications, as shown in Table VII. In the $^{32}P$ group, 25/140 patients died from vascular accidents, compared with 8/145 patients treated by busulphan.

### DISCUSSION

The significantly better results in duration of first remission and overall survival time which were obtained with busulphan treatment of PCV compared to those obtained with $^{32}P$ are in contrast to results described in the literature (Lawrence *et al.*, 1969; Osgood, 1968; Wasserman, 1971, 1976). As indicated in Table I, both treatment groups were comparable with respect to age, clinical symptoms and haematological parameters at the time of randomization. According to the trial protocol, patient follow-up and blood-volume regulations were carried out by the same physician at the same frequency for both groups.

Because the first remission duration was longer in females and in patients who were not phlebotomized previously, a correction was made for both these factors in the analysis of our data. As is shown in Tables III and IV, the differences in treatment results after statistical correction remain significantly better in the busulphan group.

Malignant complications and splenomegaly, which may occur during evolution into postpolycythaemic myeloid metaplasia (Silverstein, 1976), were observed at the same frequency in both groups.

The major difference in overall survival between the 2 groups is due to a much higher frequency of vascular accidents in the $^{32}P$ group. $^{32}P$ was given in the same average dose as that used successfully in the treatment of PCV in the literature (Lawrence, 1976; Lawrence *et al.*, 1969; Osgood, 1968; Wasserman, 1976). Retreatment was restricted to 6-month intervals and supplementary phlebotomies were carried out as necessary before and during additional $^{32}P$ treatment. Busuphan therapy could be reinstituted earlier if necessary, and gave more stable and longer lasting remissions, with less need for additional phlebotomies.

The disadvantages of using phlebotomy are the lack of effect in those cases with thrombocytosis, the difficulty in controlling the RBC volume in active cases, and the depletion of iron stores (Hutton, 1980), whereas the risk of bleeding and thromboembolism is not reduced (Wasserman, 1976). This difference in remission control, which is inherent to the mode of action of $^{32}P$, may explain the higher frequency of vascular complications in the group.

Most trials have used as chemotherapeutic agents chlorambucil, melphalan or cyclophosphamide, which require periodic cyclic administration. Busulphan-induced remissions last for months or even years without reinstitution of the drug, a result not achieved with the other alkylating agents. Busulphan should, however, never be used continuously for more than 4–6 weeks in PCV, because of the increased risk of producing persistent thrombocytopenia or leucopenia. In the dosage scheme used in this trial, busulphan was undoubtedly superior to $^{32}P$ and may therefore be regarded as the treatment of choice in polycythaemia vera.

### REFERENCES

Adamson, J. W., Fialkow, P. J., Murphy, S., Prchal, J. F. & Steinman, L. (1976) Polycythemia vera: Stem cells and probable clonal origin of the disease. *N. Engl. J. Med.*, **295**, 913.

Clarysse, A., Kenis, Y. & Mathé, G. (1976) *Cancer Chemotherapy: Its role in the treatment strategy of hematologic malignancies and solid tumors.* Heidelberg, New York: Springer Verlag.

Dameshek, W. (1951) Some speculations on the myeloproliferative syndromes. *Blood*, **6**, 372.

Eaves, C. J. & Eaves, A. C. (1978) Erythropoietin (Ep) dose-response curves for three classes of erythroid progenitors in normal human marrow and in patients with polycythemia vera. *Blood*, **52**, 1169.

Gilbert, H. S. (1975) Definition, clinical features and diagnosis of polycythemia vera. *Clin. Haematol.*, **4**, 263.

Gurney, C. W. (1970) Mechanisms underlying polycythemia. In *Regulation of Hematopoiesis.* **1.** *Red Cell Production.* Ed. Gordon. New York: Appleton Century Crofts. p. 611.

Halnan, K. E. & Russell, M. H. (1965) Polycythemia vera. Comparison of survival and causes of death in patients managed with and without radiotherapy. *Lancet*, ii, 760.

Harman, J. B. & Ledlie, E. M. (1976) Survival of polycythemia vera patients treated with radioactive phosphorus. *Br. Med. J.*, ii, 146.

Henning, K., Franke, W. J. & Strietzel, M. (1965) Eigene Erfahrungen in der Radiophosphorehandlung bei Polycythemia vera. *Z. Gesamte Inn. Med.*, **20**, 14.

Hor, G. & Pabst, H. W. (1973) Radiophosphortherapie der Polycythemia vera. *Ther. Umsch.*, **30**, 789.

Hutton, R. D. (1980) The effect of iron deficiency on whole blood viscosity in polycythemic patients. *Br. J. Haematol.*, **43**, 191.

Landaw, S. S. (1976) Acute leukaemia in polycythemia vera. *Semin Hematol.*, **13**, 33.

Lawrence, J. H. (1955) *Polycythemia. Physiology, Diagnosis and Treatment.* New York: Grune and Stratton.

Lawrence, J. H. (1976) Epilogue. *Semin. Hematol.*, **13**, 85.

Lawrence, J. H., Winchell, H. S. & Donald, W. G. (1969) Leukemia in polycythemia vera. *Ann. Intern. Med.*, **70**, 763.

Loeb, V. (1975) Treatment of polycythemia vera. *Clin. Haematol.*, **4**, 441.

Mantel, N. & Haenszel, W. (1959) Statistical aspects of the analysis of data from retrospective studies of disease. *J. Natl Cancer Inst.*, **22**, 719.

Modan, B. (1965) An epidemiological study of polycythemia vera. *Blood*, **26**, 657.

Modan, B. (1975) Inter-relationship between polycythemia vera, leukemia and myeloid metaplasia. *Clin. Haematol.*, **4**, 427.

Modan, B. & Lilienfeld, A. M. (1965) Polycythemia vera and leukemia: The role of radiation treatment. A study of 1222 patients. *Meiicine*, **44**, 305.

Osgood, E. E. (1965) Polycythemia vera: Age relationships and survival. *Blood*, **26**, 243.

Osgood, E. E. (1968) The case for $^{32}P$ treatment of polycythemia vera. *Blood*, **32**, 492.

Perkins, J., Israels, M. C. G. & Wilkinson, J. F. (1964) Polycythemia vera: Clinical studies on a series of 127 patients managed without radiation therapy. *Q. J. Med.*, **33**, 499.

Silverstein, M. N. (1976) The evolution into and the treatment of late stage polycythemia vera. *Semin. Hematol.*, **13**, 79.

Silverstein, M. N. & Lanier, A. P. (1971) Polycythemia vera, 1935–1969: An epidemiologic survey in Rochester, Minnesota. *Mayo Clinic. Proc.*, **46**, 771.

Tubiana, M., Flamant, R., Attie, E. & Hayat, M. (1968) A study of hematological complications occurring in patients with polycythemia vera treated with $^{32}P$ (based on a series of 296 patients). *Blood*, **32**, 536.

Urasinski, I. & Mysik, M. (1970) Die Therapie der Polycythaemia vera mit Busulfan (Myleran®). Bericht über die Behandlungsergebnisse von 40 Kranken. *Folia Haematol.* (*Leipz.*), **94**, 360.

Wasserman, L. R. (1971) The management of polycythemia vera. *Br. J. Haematol.*, **21**, 371.

Wasserman, L. R. (1976) The treatment of polycythemia vera. *Semin. Hematol.*, **13**, 57.